# Artificial intelligence in neuro-oncology: advances and challenges in brain tumor diagnosis, prognosis, and precision treatment

**DOI:** 10.1038/s41698-024-00575-0

**Published:** 2024-03-29

**Authors:** Sirvan Khalighi, Kartik Reddy, Abhishek Midya, Krunal Balvantbhai Pandav, Anant Madabhushi, Malak Abedalthagafi

**Affiliations:** 1 Wallace H. Coulter Department of Biomedical Engineering, Georgia Institute of Technology and Emory University, Atlanta, GA USA; 2 Department of Radiology, Emory University, Atlanta, GA USA; 3 Atlanta Veterans Administration Medical Center, Atlanta, GA USA; 4 Department of Pathology and Laboratory Medicine, Emory University, Atlanta, GA USA; 5 The Cell and Molecular Biology Program, Winship Cancer Institute, Atlanta, GA USA

**Keywords:** CNS cancer, CNS cancer

## Abstract

This review delves into the most recent advancements in applying artificial intelligence (AI) within neuro-oncology, specifically emphasizing work on gliomas, a class of brain tumors that represent a significant global health issue. AI has brought transformative innovations to brain tumor management, utilizing imaging, histopathological, and genomic tools for efficient detection, categorization, outcome prediction, and treatment planning. Assessing its influence across all facets of malignant brain tumor management- diagnosis, prognosis, and therapy- AI models outperform human evaluations in terms of accuracy and specificity. Their ability to discern molecular aspects from imaging may reduce reliance on invasive diagnostics and may accelerate the time to molecular diagnoses. The review covers AI techniques, from classical machine learning to deep learning, highlighting current applications and challenges. Promising directions for future research include multimodal data integration, generative AI, large medical language models, precise tumor delineation and characterization, and addressing racial and gender disparities. Adaptive personalized treatment strategies are also emphasized for optimizing clinical outcomes. Ethical, legal, and social implications are discussed, advocating for transparency and fairness in AI integration for neuro-oncology and providing a holistic understanding of its transformative impact on patient care.

## Introduction

Central nervous system (CNS) tumors, whether primary or secondary, exert a significant impact on global health, accounting for over 250,000 reported cases annually, marking them as a substantial global concern^1,2^. In 2022, an estimated 26,670 malignant and 66,806 non-malignant CNS tumors were diagnosed in the US population^3^. Notably, glioblastoma, a fast-growing, aggressive, and malignant type of brain tumor, emerges as a primary contributor to morbidity and mortality among adult brain tumors, exhibiting a disconcerting 6.9% 5-year survival rate and contributing to 10,000 annual deaths in the US^4,5^. These numbers highlight the current shortcomings in treating brain tumors.

Despite many clinical trials and decades of research, incurable brain tumors with grim prognoses exist, such as the diffuse midline glioma (DMG) seen in children and glioblastoma in adults^6^. This urgency highlights the need for a personalized treatment approach, which may offer the highest likelihood of cure while minimizing potential toxicity to the patient. However, the development of personalized strategies faces hurdles due to the difficulty of generalizing approaches derived from data originating in a solitary institution or a limited consortium of institutions, and restricted access to advanced technologies and clinical trials, primarily concentrated in specialized centers^7^. This becomes a critical concern, especially when contemplating the ethical ramifications associated with developing approaches based on data lacking representation across diverse demographics. Alarmingly, individuals with glioblastoma from lower socioeconomic backgrounds are less likely to undergo O6-Methylguanine-DNA-methyltransferase (MGMT) testing^8^. The absence of MGMT testing may skew predictions and contribute to late-stage diagnoses with larger and more challenging tumors. Moreover, this demographic is less frequently provided with a combination of diverse treatment modalities, leading to lower survival rates^9^.

**Fig. 1:**
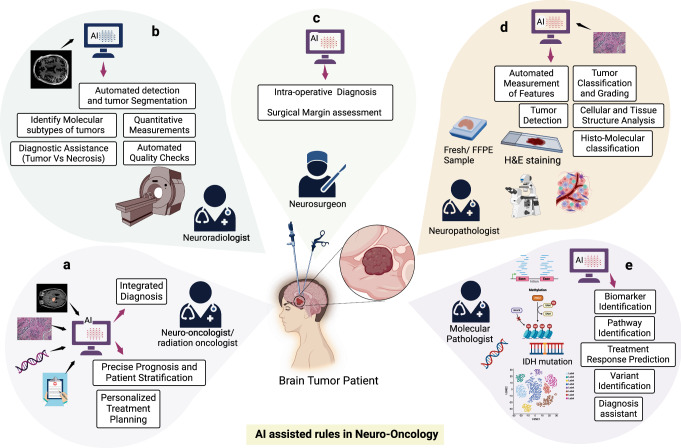
AI-empowered multidisciplinary brain tumor management. **a** AI augments the capabilities of neuro-oncologists/radiation-oncologists by enabling integrated diagnosis, offering deeper insights into the disease, facilitating precise prognosis by predicting outcomes, and assisting in patient stratification to tailor treatment plans to individual needs. **b** AI supports neuroradiologists by leveraging MRI images for automated detection and tumor segmentation, identifying molecular subtypes of tumors, providing quantitative measurements, and delivering diagnostic assistance to distinguish tumors from necrotic regions, all while ensuring automated quality checks. **c** AI aids neurosurgeons during surgery, contributing to surgical margin assessment and offering real-time diagnosis information and guidance, enhancing surgical precision and patient outcomes. **d** AI assists neuropathologists in the analysis of fresh/FFPE samples, providing automated measurement of features, aiding in tumor classification and grading, improving tumor detection, and delivering comprehensive analysis of cellular and tissue structures through histo-molecular classification. **e** Handling mutation data, single-cell information, methylation patterns, RNA sequencing, and more, AI empowers molecular pathologists by supporting biomarker identification, pathway identification, treatment response prediction, variant identification, and serving as a diagnosis assistant, streamlining the complex molecular analysis process (Created with BioRender.com).

For a patient suspected of harboring a brain tumor, the assessment typically initiates with a physical exam and neuroimaging, followed by a biopsy or tumor resection in cases where it is feasible, and subsequent histologic and molecular analyses of extracted tissue conducted through pathology. If deemed necessary, serum or cerebrospinal fluid (CSF) biomarker evaluations may also be performed^10^. Following these assessments, the clinical team must decide on optimal therapy, considering the standard of care, ongoing clinical trials, patient comorbidities, and risks of toxicity. Treatment response is monitored longitudinally through serial MRIs and, occasionally, other blood or CSF biomarkers^11^. Decisions regarding brain tumor treatment often involve multidisciplinary meetings between neuro-oncologists, neurosurgeons, neuroradiologists, molecular pathologists, and neuropathologists, underscoring the complexity of these decisions^12^ (Fig.1).

However, these steps in disease management are ridden with challenges, and errors may lead to patient morbidity and mortality^13^. The challenges include the need for precise disease diagnosis and staging to guide clinical decisions, the continuous monitoring of post-treatment disease progress, which can be complicated by signals from neighboring neural tissue, and the growing significance of identifying genotype patterns^14^. These genotype patterns have a substantial impact on tumor behavior and clinical outcomes^15^. Ultimately, the challenges of managing brain tumors arise from various factors, including the complexities of the brain, limited accessibility for accurate imaging and biopsy procedures, inherent heterogeneity of tumor biology, variable progression rates, individual variability in treatment susceptibility, and relative lack of reliable biomarkers predictive of prognosis^1,16,17^. The sensitivity of neural tissue to standard treatment modalities, including surgery, radiation, and chemotherapy, further complicates their care^18^.

Artificial intelligence (AI) shows promise as a transformative tool in neuro-oncology, currently addressing challenges across various clinical management stages. In brain tumor management, AI demonstrates its potential across diagnosis, prognosis, and treatment planning by accelerating and enhancing MRI imaging^19^, detecting abnormalities, optimizing workflows, providing accurate measurements, analyzing extensive medical imaging data, and identifying patterns not easily discernible to human observers^20^. It has significantly advanced the field by providing detailed image analysis for diagnostics, tumor grading, prognosis determination, and treatment response assessment. It also facilitates surgical and nonsurgical treatment planning^21^, accelerates drug discovery^22^, and facilitates recurrence monitoring. AI tools can be incorporated into clinical trials, aiming to improve patient outcomes and may provide the path toward personalized therapy^15,23^. In clinical neuroimaging, AI plays a crucial role in tasks such as identifying tumor boundaries and types, refining pre-therapeutic planning, and assessing post-therapeutic responses^24^. The capacity for AI to process extensive datasets offers a transformative approach to precision medicine, potentially addressing commonly encountered pain points at all steps of the patient care experience^25–27^ (Fig. 1). Additionally, it holds promise in ameliorating global healthcare disparities by providing democratized access to diagnostic, prognostic, and therapeutic strategies^28,29^.

Recently, there has been growing exploration of integrating AI tools into radiological and pathological workflows, suggesting potential advancements in neuro-oncology^30,31^. In brain tumor analysis, AI serves as a comprehensive framework that encapsulates machine learning (ML) and deep learning (DL) techniques, computer vision (CV), and their integration into Computational Biology. ML algorithms within AI contribute to pattern recognition in imaging and genomic data, while DL, a subset of ML, excels in intricate feature extraction. Computer vision, whether through classical image processing techniques or advanced DL methods, interprets visual data for precise medical image analysis. Computational biology leverages AI, ML, and DL to analyze extensive biological datasets, aiding in understanding the genetic and molecular aspects of brain tumors (Supplementary Table 1). The synergy among these techniques enhances the depth and accuracy of brain tumor characterization, influencing diagnosis, prognosis, and treatment planning.

In conducting this review, a comprehensive literature search was conducted across several electronic databases. The search was focused on recently published articles, with an emphasis on studies related to AI applications in brain tumor diagnosis, prognosis, and precision treatment. Our search strategy prioritized peer-reviewed articles, systematic reviews, meta-analyses, and landmark studies in the field. This narrative review provides a comprehensive understanding of AI’s pivotal role in managing primary malignant brain tumors, focusing on gliomas. It explores AI applications in brain tumor diagnosis, prognosis, treatment planning, and predictive analytics. Addressing the multifaceted nature of AI in neuro-oncology, we discuss biomarkers, ethical implications, innovative methods, and challenges, including considerations for racial and sex-specific differences within AI applications and efforts to address disparities in current work limitations. What sets our review apart is its explicit focus on integrating AI in radiology, pathology, and genomics for comprehensive brain tumor analysis. Unlike previous papers, our review emphasizes the convergence of AI applications across radiology, pathology, and genomics, providing a holistic approach to brain tumor diagnostics, prognostics, and treatment planning. While many prior reviews have discussed AI in neuro-oncology broadly, they often lack a specific emphasis on the synergistic integration of AI across these critical domains. Concentrating on diagnostic, prognostic, and treatment planning within the imaging domain, our paper not only explores the latest advancements in AI tailored to pathology, radiology, and genomics but also addresses the gaps left by previous reviews in fully comprehending the interconnected roles of these disciplines in brain tumor management. This focused approach contributes a unique perspective, detailing AI’s transformative role in refining imaging-based diagnoses, prognoses, and treatment planning, which were not thoroughly covered by the broader, less-specialized reviews in the field.

### Data types and datasets for brain tumor analysis

Brain tumor analysis relies on a range of data types that are effectively utilized by AI algorithms to unveil crucial characteristics. Key data categories encompass imaging data, genomic data, and clinical data. Medical imaging techniques, such as MRI and CT, offer the opportunity to extract intricate visual features about tumor size, location, morphology, and texture. The current standard for brain tumor imaging involves multi-parametric MRI, including sequences like pre- and post-contrast T1-weighted, T2-weighted, fluid-attenuated inversion recovery (FLAIR), diffusion-weighted (DWI), and susceptibility-weighted imaging (SWI) as commonly obtained sequences. High-volume neuro-oncology centers often incorporate additional techniques like MR spectroscopy and perfusion imaging^11,32^. Beyond standard imaging, radiomics extracts quantitative features, while histopathological data, derived from biopsies or surgical resections, encompasses tumor cell morphology and tissue architecture. Genomic data, derived from DNA and RNA sequencing, transcriptomic analysis, and methylation analysis, aids in classifying subtypes and predicting tumor aggressiveness^33^. Moreover, prominent molecular biomarkers play a pivotal role in discriminating between brain tumor subtypes^1,9,34^. These include mutations in IDH for astrocytomas and oligodendrogliomas, TERT promoter mutations for glioblastomas, EGFR amplification for glioblastomas, gain of chromosome 7 and loss of chromosome 10 for glioblastomas, ATRX mutations for astrocytomas, MGMT promoter methylation for glioblastomas, co-deletion of 1p and 19q chromosomes for oligodendrogliomas, and distinct molecular subtypes for medulloblastoma (MBs)^9^ (See Tables 2 and 3). Lastly, Clinical data, inclusive of patient history, medical records, and treatment responses, contributes to a comprehensive diagnostic profile, with outcomes data serving as a crucial reference for survival prediction models (see Table 1).

**Table 1:** Overview of data types in brain tumor analysis

Radiomics Data	Description: radiomics involves extracting quantitative features from medical imaging data, such as MRI or CT scans.
	Purpose: it aims to capture and analyze the texture, shape, and intensity patterns of tumors, providing additional information for diagnosis, prognosis, and treatment planning.
Pathological Data	Description: pathological data involves the examination of tissue samples from the tumor through biopsy or resection.
	Purpose: pathological analysis helps determine the tumor’s histological type, grade, and molecular characteristics, aiding in treatment decisions.
Genomic Data	Description: genomic data involves analyzing the genetic makeup of tumors through techniques like next-generation sequencing (NGS).
	Purpose: it provides insights into genetic mutations, alterations, and expression patterns, guiding personalized treatment approaches.
Clinical Data:	Description: clinical data encompasses patient-related information, including demographics, medical history, and treatment records.
	Purpose: integration of clinical data with other datasets aids in understanding patient-specific factors influencing tumor behavior and treatment responses.
Multimodal Data (MRI and CT)	Description: multimodal data combines information from different imaging modalities, commonly MRI and CT scans.
	Purpose: combining data from multiple modalities enhances the overall understanding of the tumor’s characteristics, offering a more comprehensive view.
Multi-parametric MRI	Description: multi-parametric MRI involves acquiring images using various sequences such as T1-weighted, T2-weighted, FLAIR, DWI, and SWI.
	Purpose: different sequences provide diverse information about the tumor’s structure, function, and blood supply, aiding in accurate diagnosis.
MR Spectroscopy and Perfusion Imaging	Description: MR spectroscopy assesses the chemical composition of tissues, while perfusion imaging measures blood flow.
	Purpose: these techniques provide information on metabolic activity and vascularization, assisting in tumor characterization.
Next-generation Sequencing (NGS)	Description: NGS is a high-throughput sequencing technology that analyzes DNA, RNA, or both.
	Purpose: in brain tumor analysis, NGS helps identify genetic mutations, fusions, and variations, guiding targeted therapies.
Circulating Tumor DNA (ctDNA) Analysis:	Description: ctDNA analysis involves detecting tumor-derived genetic material circulating in the bloodstream.
	Purpose: it enables non-invasive monitoring of tumor dynamics, treatment response, and the emergence of resistance.

Each data type is described along with its purpose, elucidating its role in enhancing diagnosis, prognosis, and treatment planning for brain tumors.

**Fig. 2:**
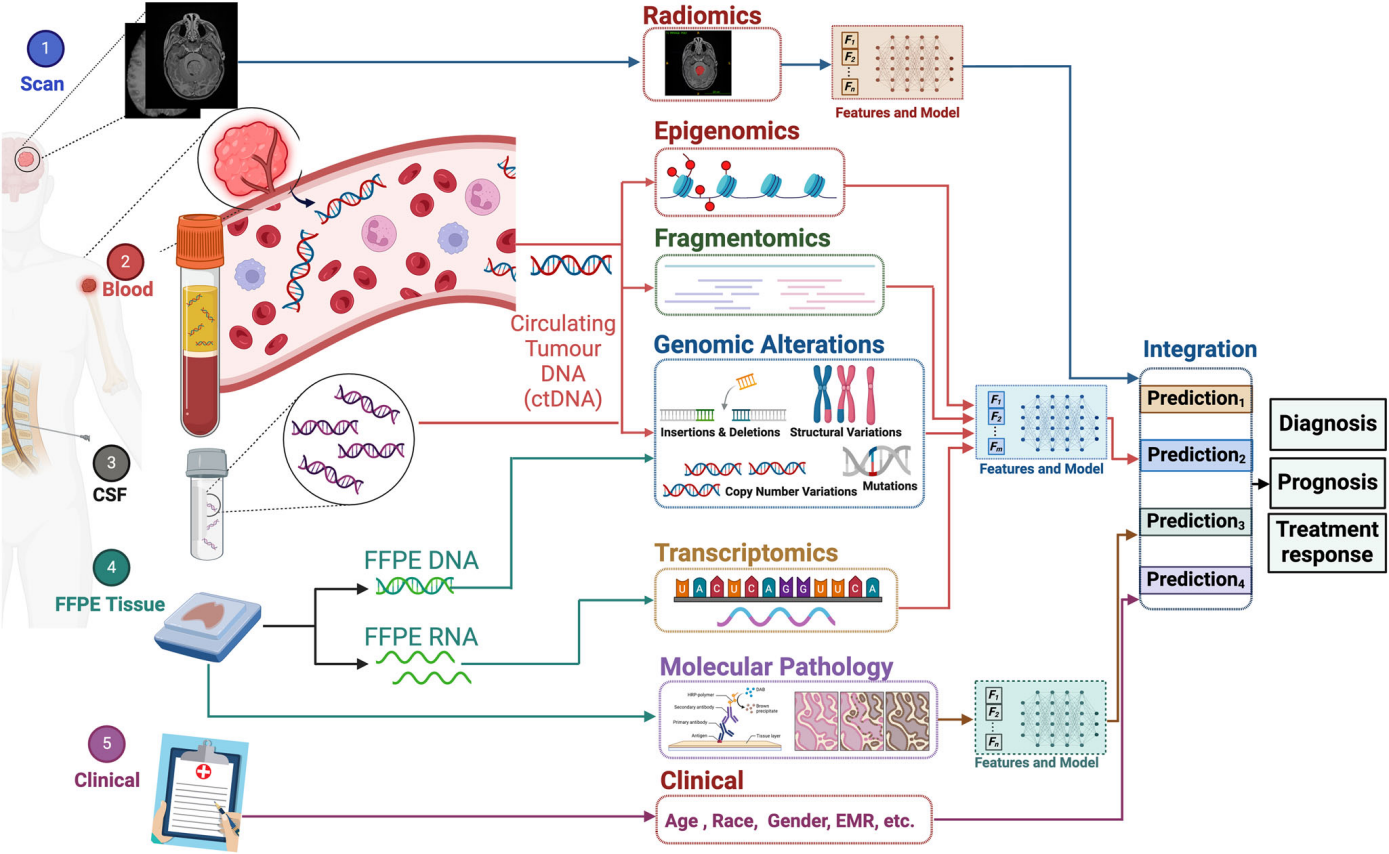
Multimodal integration for enhanced diagnosis, prognosis, and treatment response prediction in brain tumors. Shown is the structural framework of a multimodal integration method designed to improve brain tumor management. The process involves the assimilation of data from five different sources, each contributing unique information. From MRI scans, radiomic data is generated. This data includes segmented MRI images achieved through AI-driven segmentation techniques, providing information about the tumor’s spatial characteristics. Blood samples yield ctDNA, allowing for the extraction of epigenomic, fragmentomic, and genomic alterations that inform the molecular landscape of the tumor. CFS samples provide cell-free DNA (cfDNA), offering genomic alteration information and contributing to a comprehensive understanding of the tumor’s genetic profile. Formalin-fixed paraffin-embedded (FFPE) tissue samples provide transcriptomic and molecular pathology data, offering information about gene expression and cellular structure. Clinical information such as age, race, gender, and electronic medical records (EMR) data supplement the molecular and imaging data, enriching the patient’s profile. For each of these modalities, feature extraction is performed, generating a set of informative characteristics. Subsequently, predictive models are applied to each dataset to estimate key outcomes related to diagnosis, prognosis, and treatment response. In the late multimodal integration, the predictions from these distinct models are fused to improve performance and precision. By synthesizing information from diverse sources and modalities, the integrated approach enhances the reliability and accuracy of neuro-oncological diagnosis, prognosis, and treatment response prediction (Created with BioRender.com).

In addition to more standardly employed data types, innovative approaches like liquid biopsies have emerged for the early detection of brain tumors^35^. Circulating tumor DNA (ctDNA) analysis, a non-invasive method, monitors tumor mutations and genetic changes through fragments of tumor DNA in the bloodstream^36,37^. The integration of these diverse data types and advanced technologies enables a new era of accurate, minimally invasive, and effective approaches for diagnosing and treating brain tumors, overcoming the limitations of conventional diagnostic methods. The integration of multiple data sources through multimodal data fusion enhances analyses accuracy by offering a more comprehensive view of the tumor’s characteristics and behavior^38^(Fig. 2). A concise overview of each data type, including its description and purpose, is provided in Table 1.

In addition to the institutional datasets, numerous public datasets play a crucial role in evaluating AI-based algorithms for brain tumor diagnosis, prognosis, and treatment planning. Tailored to diverse research needs, these datasets cover various aspects of the disease. The Cancer Imaging Archive (TCIA) is notable among general brain tumor datasets, offering a comprehensive repository of medical imaging data, including MRI, CT, and PET scans for various tumor types^39^. The MICCAI BraTS Challenge provides standardized brain tumor segmentation datasets annually, ideal for assessing algorithms focused on precise tumor delineation^40^.

For specific tumor types, resources such as the NCI TARGET dataset include dedicated sections for glioblastoma (TCGA-GBM) and lower-grade gliomas (TCGA-LGG)^41^. Additional platforms such as the open data alliance and the NCI data commons offer open-access datasets across scientific domains, including medical and brain tumor datasets^42,43^. Selecting the most appropriate dataset depends on factors like tumor type, imaging modality, data type (MRI, CT, PET, etc.), availability of ground truth annotations, and data size, allowing researchers to align their choice with specific research interests for AI-driven investigations into brain tumor diagnosis and treatment planning.

### Advancements in AI-enhanced preprocessing for precision brain tumor analysis

In brain tumor analysis, AI has addressed the challenges of navigating brain anatomy and tumor variability and significantly enhanced crucial preprocessing steps for accurate diagnosis, prognosis, and treatment planning. Addressing issues of spatial consistency, AI-powered algorithms, such as those integrated into the BrainNet viewer, correct artifacts and distortions in MRI images^44^. This correction facilitates more precise tumor localization and segmentation, which is crucial for effective brain tumor analysis.

Moreover, AI streamlines the intricate process of tumor localization with remarkable accuracy, as demonstrated by algorithms evaluated on the BraTS dataset^40^. Notably, some AI-empowered methods have achieved high accuracy in localizing tumors, thereby enhancing efficiency for radiologists and reducing the potential for human error^45,46^. Image segmentation via CNNs, which are adept at uncovering complex patterns from data, has emerged as a powerful tool^47–51^. AI-driven algorithms, including the nnU-Net algorithm^52^, demonstrate exceptional proficiency in automating the crucial task of segmenting normal tissues in medical images. This segmentation is vital for tumor analysis, assisting radiologists in identifying areas to avoid during radiation therapy or surgery^52^. In recent developments, the federated learning framework has demonstrated comparable or superior results in the automated segmentation of rare pediatric tumors from MRI images. This approach leverages data from diverse institutions while ensuring the utmost confidentiality of patient information^24^.

The integration of multimodal data enhances detection efficacy by tapping into diverse information sources^53^. The landscape of DL introduces innovative architectures, with some notable exemplars, including the 3D U-Net^46^, DeepMedic^54^, and V-Net^55^. The 3D U-Net, designed to excel in segmenting 3D images of glioblastoma, and recognized for its straightforward training and consistent effectiveness, achieves remarkable results in brain tumor segmentation. DeepMedic^54^, known for its robustness in managing noise and artifacts, stands as a strong competitor to the 3D U-Net, trained on glioma images. The V-Net, a nascent innovation designed to accurately segment volumetric medical images, establishes its prowess in segmenting both 2D and 3D MRI images^55^. Overall, AI’s role in these preprocessing steps empowers radiologists to conduct brain tumor analysis with heightened precision and efficiency.

## AI in brain tumor diagnosis

Brain tumor diagnosis involves the identification and characterization of abnormal growths or masses within the brain, utilizing various medical imaging, pathological, and clinical methods to determine the nature, location, and characteristics of the tumor^55^. Brain tumors vary based on their origin, location, histology, malignancy, and patient age, and categorizing them is crucial for diagnostics, prognosis, and treatment planning^55^. These tumors consist of diverse subtypes, each characterized by distinct cellular origins and histological features. While pediatric-type low and high-grade gliomas and MB are most common in children, glioblastomas, diffuse gliomas, and meningiomas predominate in adults^56^. Given their significance, the predominant focus of research has been on glioblastoma and other diffuse glioma in adults and MB, and pediatric type low- and high-grade glioma in children^57,58^. Accurate identification of the tumor subtype empowers clinicians to customize diagnostic methods, predict disease behavior, and inform targeted therapies^59^. Unique genetic and histological signatures associated with different tumor types provide information about aggressiveness and treatment responses, guiding the selection of imaging modalities, biomarker assessments, and treatment plans^60^.

Brain tumors are often identified on CT performed in the emergency room setting, prior to being further characterized via MRI, and are definitively diagnosed via histopathologic examination^56^. Standard of care in neuropathology now includes molecular and genetic testing for many tumor types, guided by initial histologic findings^57^. Current diagnosis methods face challenges^58–60^ such as early detection due to tumor concealment^61^, imaging limitations^20^, and issues of visualizing small or deep-seated tumors^62,63^, difficulties in distinguishing tumor types^64^, invasive procedures with associated risks^65^, and the heterogeneity of brain tumors^66^. These approaches are hindered by time-consuming processes, limited accessibility, and interpretation variability among experts, highlighting the need for advanced AI-based methods^16,67^.

AI can add value at all steps of tumor diagnosis, with the majority of current studies attempting to create predictive models trained using imaging data, pathology data, or both data modes combined^68^. The integration of AI models in brain tumor diagnosis shows potential, particularly in distinguishing between glioma and solitary brain metastases using quantitative image analysis methods^69,70^. Neuroimaging provides a unique glimpse into the unaltered tumor in its entirety, while pathologic analyses provide a more in-depth look at the cellular and molecular features of the tumor. ML, as a key element of AI, is contributing to advancements in brain tumor diagnostics by enhancing accuracy, expediting image analysis, enabling early detection, and improving differentiation between tumor types. Recent progress in CV, ML, and DL holds the potential for addressing challenges and improving patient care in brain tumor diagnosis^45^.

### AI-empowered radiology, and histology-based diagnostic methods

Radiology- and histology-based brain tumor diagnosis involves extracting quantitative features from medical images such as MRI scans or H&E WHI to capture tumor morphology, texture, and spatial relationships. Traditional feature extraction approaches such as texture analysis (Gray-level co-occurrence matrix (GLCM), Gray-level run-length matrix (GLRLM), and Haralick^71,72^), Shape analysis^73^, Intensity analysis^74^, Wavelet-based analysis^75^, are complemented by newer approaches. Then, ML and DL construct predictive models, enabling a personalized, data-driven approach to diagnosis^31,76,77^. Common techniques comprise CNNs, RNNs, vision transformers, generative adversarial networks (GANs)^78^, support vector machines, and random forests^77^ (Supplementary Table 1). Models generated from multiple sequences, such as mpMRI have been shown to be more accurate when compared to single sequence models^79^ for tumor detection, assessing grades, and guiding treatment planning^80^.

The histology-based diagnosis methods, aligned by the 2021 WHO (World Health Organization) CNS classification book, are central to brain tumor pathology. The histology-based analysis encompasses various molecular techniques leveraging histological data to enhance brain tumor diagnosis. Methylome profiling, a recently influential technique using AI/ML-based classifiers, has become an influential technique for categorizing and diagnosing brain tumors^81^. While the 2021 WHO classification supports the use of methylome classifiers for various brain tumors either as essential or desirable criteria, there’s ongoing debate over the best method and the limited accessibility of diagnostic tests^34^. DeepGlioma^82^, an AI-based diagnostic screening system, offers rapid results (<90 seconds) by streamlining the molecular diagnosis of GMGs using stimulated Raman histology (SRH) images. This innovative system has been developed and validated on a multicenter cohort, highlighting its potential for rapid and accurate brain tumor diagnosis^82^.

Furthermore, innovative approaches utilizing radiomics on MRI perfusion scans demonstrated the ability to predict IDH mutations, providing valuable information for diagnosis and treatment planning^83^. Terahertz spectroscopy has been explored as a non-invasive technique for predicting IDH mutations in glioma tissue samples, presenting a promising alternative to existing methods^84^. Moreover, advanced analysis techniques applied to 18F-FET PET/CT scans have enabled the prediction of both glioma grade and IDH mutation status in untreated patients^85^. Notably, a deep learning imaging signature (DLIS) has been developed, offering accurate prediction of 1p/19q co-deletion in diffuse lower-grade gliomas through pre-operative MRI scans, presenting a non-invasive alternative with significant diagnostic potential^86^.

The Integration of immunohistochemistry, methylation profiling, chromosomal microarray, scRNA-seq^87^, and NGS, with histology-based analysis could further enhance brain tumor diagnosis^81,88^. While conventional approaches, utilizing imaging, tissue biopsies, and genetic testing, confidently identify many brain tumors by combining histology with specific genetic changes, exceptions exist, such as high-grade astrocytoma with piloid features, introduced in the 2021 WHO classification^88,89^. This particular condition demands methylome profiling for diagnosis^89^, but its rarity suggests that methylome classifiers are best suited for specific cases with atypical clinical and pathological presentations. A recent deep learning method named “Sturgeon,” can rapidly and accurately classify CNS tumor types during surgery using sparse methylation array data obtained from nanopore sequencing produced during surgery. It classifies CNS tumors within 40 minutes after starting sequencing, with an accuracy of 72% in real-time surgical settings^90^. This method allows surgeons to make more informed decisions about the extent of resection, potentially reducing the risk of complications and improving patient outcomes.

## AI in brain tumor prognosis

Prognosis in neuro-oncology involves estimating disease progression for an individual, considering treatment planning, disease stage, and site^91^. Key metrics are overall survival (OS), and progression-free survival (PFS), crucial for assessing prognosis and guiding treatment^92^. However, conventional methods relying on disease stage and clinical variables face limitations, including interpretational complexities, biases, and the need for extensive datasets. Achieving precision for personalized care in predicting recurrence and survival remains challenging with conventional methods.

In brain tumor care, AI plays a pivotal role in advancing prognostic capabilities. ML and DL techniques are increasingly being harnessed to predict OS, and PFS, leveraging features extracted from pre-treatment imaging data. Noteworthy studies, including radiomic signatures from T1 and FLAIR MRI scans of glioblastoma patients^69^, and T1, T2, and FLAIR scans from treatment-naïve patients, show significant promise in predicting PFS and OS^93,94^. The AI models outperform routine clinical variables and demonstrate excellence when combined with clinical attributes in glioblastoma patients^95,96^. Remarkably, models based on T2-weighted MRI^97^ and radiomic features from peritumoral edema reveal associations with survival outcomes, site of recurrence, and molecular subtype^98^, especially in glioma^97^, and glioblastoma patients^99^. DL-based models are created to identify tumors and forecast the site of recurrence, sometimes before radiologists can detect it^100^. These models, using various imaging methods, highlight AI’s exceptional predictive capabilities^97,99^ (Table 2).

**Table 2:** AI-Based prognostic models in brain tumor studies: patient cohorts and feature extraction strategies

Study	Patient cohort	Tumor type	Treatment status	Feature extraction
Kickingereder et al.^93^	119 patients, T1 and FLAIR	Glioblastoma	Possibly treatment-naive	Handcrafted radiomic features
Prasanna et al.^94^	65 patients, T1, T2, FLAIR	Glioblastoma	Treatment naive	Handcrafted radiomic features
Kickingereder et al.^95^	181 patients, T1, T2, FLAIR	Glioblastoma	Treatment naive	Handcrafted radiomic features
Kim et al.^96^	83 patients, T1 and FLAIR	Glioblastoma	Pre-operative	Handcrafted radiomic features
Li et al.^97^	652 patients, T2 scan	Glioma	Pre-operative	Handcrafted radiomic features
Lyer et al.^98^	88 patients, T1	Medulloblastoma	NA	Handcrafted radiomic features
Long et al.^99^	22 patients, T1 and FLAIR scan	Glioblastoma	Pre-operative scan	Handcrafted radiomic features
Zhou et al.^100^	FLAIR and T1 scan	NA	Both presurgical and post-surgical	Deep learning
Zheng et al.^30^	410 patients, Single-cell RNA seq and spatial transcriptomics	Glioblastoma	NA	Deep learning

Furthermore, Intra-tumoral heterogeneity and cell-state plasticity have been identified as key drivers for the therapeutic resistance of glioblastoma^30^. Spatial transcriptional profiles and prognosis from histology images were predicted using this DL framework, shedding light on the potential of AI in unraveling complex aspects of tumor behavior^30^. Additionally, the identification of IDH mutations has been leveraged to guide prognosis, while the definition of glioblastoma has been refined through the analysis of TERT promoter, EGFR amplification, gain of chromosome 7, and loss of chromosome 10. Additionally, H3F3A has emerged as a key marker for aggressive pediatric tumors^101^.

## AI in brain tumor therapeutic management

In addition to adding value to both diagnostic and prognostic capabilities, AI has been used for improving brain tumor treatment planning and treatment response assessment^102^. It transforms therapeutic approaches and enhances precision by aiding in the identification and characterization of brain tumors. It guides clinicians in determining the most suitable treatment strategies for individual patients. This multifaceted process involves diverse techniques, including imaging, clinical assessments, biopsies, and molecular analyses, for precise determination of tumor presence, type, location, and extent.

AI-based methods excel in predicting therapy responses, enabling improved treatment planning across various cancers^103^. Novel approaches, such as predicting responses to gamma knife radiosurgery for metastatic brain tumors using radiomic features^104^ from contrast-enhanced T1 and FLASH scan and utilizing predictive models based on pre-treatment ADC maps for forecasting responses to radiation therapy, showcase AI’s efficacy^104^. Integrated models, combining radiomics with clinical attributes, effectively assess radiotherapy responses for patients with brain metastasis from primary breast and lung cancer across multicenter patient cohorts^105^. Spatial heterogeneity analysis of peritumoral edema (ED) in glioblastoma aids in identifying high-risk habitats within ED, leading to enhanced treatment planning^106^. While identifying the crucial marker MGMT for temozolomide (TMZ) resistance in glioblastoma patients presents challenges, AI-based radiomic methods emerge as predictors of both MGMT status and TMZ response, providing valuable insights for informed treatment decisions^101,107^ (Table 3).

**Table 3:** AI-enhanced therapeutic management of brain tumors: insights from studies and treatment modalities

Study	Data type	Disease	Treatment	Method
Kawahara et al.^104^	88 patients, T1 MRI	renal cell or melanoma cancer as the primary disease and metastasis in brain	Gamma knife radiosurgery	Handcrafted radiomic features
Wang et al.^105^	258 patients, T1 MRI	NSLC as primary and me metastasis in brain	whole-brain radiotherapy	Handcrafted radiomic features
Yang et al.^106^	122 patients, T1 and T2-FLAIR (presurgical)	Glioblastoma	Surgery	Handcrafted radiomic features
Do et al.^107^	T1, T2, T2-FLAIR	Glioblastoma and Giloma	NA	Handcrafted radiomic features

## Integrative multimodal and multiscale analysis

In multimodal and multiscale approaches, the hope is for a more comprehensive understanding of brain tumors through the integration of genomics, pathomics, and radiomics data. Genomics, especially through techniques like NGS, takes a prominent role in unraveling the genetic landscape of brain tumors, providing information into their genomic alterations^88^. Molecular subtyping and biomarkers identified play a critical role in personalized precision medicine, impacting early detection, prognosis, and treatment response prediction. This integrative approach, when combined with clinical data, advances our comprehension and lays the groundwork for tailored treatments targeting specific genetic alterations.

Complementing this genomic foundation, multimodal imaging techniques such as MRI, CT, and PET contribute a rich layer to the integrative tapestry, albeit with challenges of cost and time^108^. When fused with clinical expertize and other diagnostic data, multimodal imaging significantly enhances diagnostic accuracy. The integration of genomics and radiomics, facilitated by AI, emerges as a revolutionary force in understanding and treating brain tumors.

Furthermore, AI’s advancements extend beyond unimodal predictions, ushering in an era of multimodal prognostic and treatment approaches. These multiscale, multimodal approaches extract features from diverse data sources, including radiomic images and multimodal imaging, resulting in a more comprehensive and accurate understanding of the disease trajectory^100^ (Fig. 2).

## Challenges and limitations of AI in brain tumor diagnosis, prognosis, and treatment

Despite the successful integration of AI models in different steps of brain tumor management, challenges persist. These challenges include restricted access to high-quality data, concerns regarding the interpretability and explainability of DL models, and the need for generalizability across diverse populations and tumor types^109^. The reproducibility of radiomic-based features across different institutes faces challenges due to variations in image acquisition parameters, including machines, models, and contrast amounts^110^. Particularly, achieving reproducibility is more complex in MR radiomics compared to CT radiomics^111^. To standardize radiomics, the introduction of the radiomic quality score (RQS) has been pivotal^112^. However, despite the importance of validation of the AI method in neuro-oncology using external dataset, only 29.4% of original studies included external validation^113^.

Additionally, in brain tumors management, it becomes evident that racial disparities introduce intricate dynamics shaped by race, socioeconomic variables, and geographical influences^114^. This complexity extends to various aspects, including recommendations for brain tumor surgery^115^, emphasizing the importance of addressing such disparities in AI-based methods throughout the spectrum of brain tumor management to advance cancer care. Moreover, disparities in brain tumor rates and outcomes, particularly in glioblastoma, manifest differently between males and females^116^. This underscores the necessity for AI-based approaches to factor in sex-related influences across incidence, survival, tumor biology, genetics, treatment response, and prognosis. The key advantages of these models lie in offering enhanced predictions for personalized treatment and the potential for early detection by accounting for gender-specific characteristics.

### Ethical, legal, and social implications of AI in brain tumor management

The integration of AI in brain tumor diagnosis, prognosis, and treatment raises critical ethical, legal, and social considerations^117^. Key ethical concerns include ensuring patient privacy through robust data privacy measures, obtaining informed consent, addressing algorithmic fairness, and promoting transparency in AI algorithms and accountability to build and maintain patient trust^28^. Innovations like federated learning aim to tackle the privacy challenge in AI by enabling collaborative model training among multiple parties without the need to share raw data^24^. Ethical imperatives extend to addressing biases and ensuring equitable access. Legal considerations, encompassing liability for AI-generated errors, medical malpractice standards, and regulatory compliance, underscore the need for robust legal frameworks. Collaborative efforts involving policymakers, regulatory bodies, and legal experts are crucial to clarify responsibilities, protect patient safety, and foster responsible AI development. Social implications, such as impacts on patient-doctor relationships, patient empowerment, and healthcare disparities, require careful consideration. AI has the potential to empower patients by providing personalized information and enabling shared decision-making^117,118^. However, the equitable access and affordability of AI-driven healthcare need to be addressed to avoid exacerbating existing disparities^117,118^.

## Discussion

This review highlights the transformative impact of AI in brain tumor management, signifying a paradigm shift in healthcare that addresses longstanding challenges. AI’s proficiency in ML and DL techniques, specifically in image segmentation, spatial consistency, and prediction, enhances precision in identifying and characterizing brain tumors. This precision contributes to improved diagnostics, prognosis, and personalized treatment planning. The seamless integration of diverse data types, from medical imaging to genomics, along with clinical history, enables a holistic understanding of tumor characteristics, shaping prognosis and personalized treatment plans. AI’s potential to empower clinicians with real-time monitoring, enhanced treatment planning, and optimization is emphasized, promising improved patient outcomes.

The prognostic capabilities of AI-based models surpass routine clinical variables, providing superior predictive accuracy and refining survival predictions^30^. The integration of AI in predicting treatment response, survival time, and site of recurrence is a significant advancement, enabling precise, personalized therapies tailored to individual tumor characteristics and patient-specific data. AI-driven diagnosis not only facilitates real-time monitoring but also improves treatment planning and optimization. This section highlights AI’s potential to deliver more precise, personalized, and effective interventions, contributing to enhanced patient outcomes. Clinical decision support systems, empowered by AI, not only provide evidence-based treatment recommendations but also contribute to ongoing research by generating novel insights and biomarkers.

Ethical considerations in the integration of AI in brain tumor diagnosis, prognosis, and treatment are acknowledged, covering data privacy, algorithmic fairness, legal liability, and social implications. Robust legal frameworks and collaborative efforts such as federated learning are deemed necessary to address these challenges for responsible AI development and societal acceptance in healthcare. Despite challenges such as data collection costs and interpretational complexities, AI integration holds substantial promise, offering prospects for precise and personalized patient care in the future. However, workforce changes and training may be necessary to effectively integrate AI technologies into healthcare settings. The ethical and societal acceptance of AI in healthcare depends on transparent communication, addressing privacy concerns, and promoting fair and inclusive practices.

Overall, AI extends its influence into treatment planning, revolutionizing therapeutic strategies and significantly contributing to improved patient outcomes. The integration of AI into the treatment landscape holds promise for personalized and effective interventions in neuro-oncology.

### A vision for the future: a spectrum of approaches

The integration of AI in brain tumor diagnosis, treatment, and prognosis has seen significant progress, yet there are still gaps and promising future directions to explore. Multimodal data integration^108^, real-time monitoring, diagnosis, and adaptive treatment strategies hold the potential to enhance diagnostic accuracy and treatment outcomes^90^. AI can play a critical role in long-term prognostication and survivorship care planning, aiding in treatment decision-making. Bridging the gap between clinical practice and research through data-sharing networks can accelerate AI model development and validation. Transparency and interpretability of AI models are essential for gaining trust and acceptance in clinical settings^119,120^. Ethical considerations and human-centered design principles must be prioritized to ensure responsible and patient-centric AI integration. By addressing these aspects, AI has the potential to revolutionize brain tumor care and improve patient outcomes^120^.

Envisioning the future of brain tumor analysis, DL stands at the forefront, but various techniques beckon exploration based on specific applications and data availability. Improved imaging methods, such as functional magnetic resonance imaging (fMRI)^121^ and diffusion tensor imaging (DTI)^122^, provide more precise tumor characterization, despite challenges like sensitivity to noise, head motion, magnetic field distortions, and computational expenses^123,124^. Computational advancements, notably CNNs and Transformer-based models, enhance accuracy in the detection and classification of brain tumors^125–127^. Multimodal data fusion^108^, encompassing MRI, CT, and PET scans^38^, along with transfer learning using pre-trained models from vast image datasets, addresses the challenge of limited labeled medical data^77^ (Supplementary Table 1).

Graph-based methods leverage intricate brain region relationships^128^, with graph neural networks (GNNs) and graph-based convolutional networks (GCNs) illuminating the path of modeling brain connectivity and uncovering tumor-associated anomalies^129^. Radiomics and feature engineering extract an array of quantitative features from medical images, with ML algorithms illuminating patterns and correlations. Explainable AI (XAI), a pivotal facet, ensures algorithm transparency and interpretability, a cornerstone in medical applications^130,131^. Recent endeavors have gravitated toward devising AI models with explicable outputs, fostering clinicians’ comprehension of decision-making processes, and thus nurturing trust in automated brain tumor detection and classification. Data augmentation and synthesis techniques, encompassing image rotation, scaling, flipping, and the ingenuity of GANs, fortify the training dataset’s robustness^78,127^. Additionally, AutoML and hyperparameter optimization tools streamline the optimization of architecture and hyperparameters in brain tumor detection algorithms, culminating in more efficient and precise models.

Collaborative platforms and datasets, such as federated learning^24,132^ burgeoning repositories of meticulously annotated brain tumor data, expedite algorithmic training, evaluation, and innovation while catalyzing benchmarking efforts. The realm of real-time detection, propelled by advances in hardware such as graphics processing units (GPUs) and field-programmable gate arrays (FPGAs), unlocks the potential for real-time processing of medical images^90,133^. Such real-time algorithms hold the promise of streamlining clinical workflows and elevating patient care. The recent utilization of Large Language Models (LLMs) in neurological research demonstrates a remarkable capacity to analyze diverse data sources, offering significant contributions to early diagnosis, patient support, and clinical assistance. Noteworthy challenges, including concerns related to data privacy and biases, highlight the imperative for collaborative endeavors to ensure the responsible development of LLMs in neurology^134^ (Supplementary Table 1).

## Conclusions

We explored the transformative applications of AI, including CV, ML, and DL, in managing brain tumors. AI shows significant promise in diagnosis, prognosis, and treatment planning by effectively detecting and classifying brain tumors from medical images. Through radiomic, pathomic, and genomic analyses, AI contributes to precise tumor characterization. In treatment, AI plays a crucial role in planning, optimization, and response prediction, supporting personalized recommendations and real-time monitoring. The integration of AI-driven approaches aligns with precision medicine and patient-centered care. However, the adoption of AI in brain tumor management requires careful consideration of ethical, legal, and social implications, addressing concerns related to data privacy and healthcare disparities.

Future directions include bridging research gaps, exploring LLM models, multimodal data integration, and advancing real-time monitoring. AI models trained on diverse datasets hold promise for predicting treatment responses and improving patient outcomes. Ongoing development and fine-tuning are essential for uncovering the full potential and challenges in the clinical management of brain tumors, positioning AI as a valuable tool in research and practice.

### Supplementary information

supp tables
