# Supplementary material for: Artificial intelligence in neuro-oncology: advances and challenges in brain tumor diagnosis, prognosis, and precision treatment
Source: NPJ Precis Oncol. 2024 Mar 29;8:80. doi: 10.1038/s41698-024-00575-0 (PMC10980741; doi:10.1038/s41698-024-00575-0)
Supplement: Supplementary file 1 — supp tables [file 41698_2024_575_MOESM1_ESM.pdf]

Artificial intelligence in neuro-oncology: Advances and challenges in brain tumor diagnosis, prognosis, and precision treatment

Sirvan Khalighi[1], Kartik Reddy[2], Abhishek Midya[1], Krunal Balvantbhai Pandav[1], Anant Madabhushi[1,4,*], Malak Abedalthagafi [3,5,*]

[1]Wallace H. Coulter Department of Biomedical Engineering, Georgia Institute of Technology and Emory University, Atlanta, GA, USA

[2]Department of Radiology, Emory University, Atlanta GA, USA

[3]Department of Pathology and Laboratory Medicine, Emory University, Atlanta GA, USA

[4]Atlanta Veterans Administration Medical Center, Atlanta GA, USA

[5]The Cell and Molecular Biology Program, Winship Cancer Institute, Atlanta GA, USA

***Correspondence**:
E-mail: Anant.Madabhushi@emory.edu , Malak.althgafi@emory.edu

Supplementary Materials

Supplementary Table 1. Key Concepts in AI for Neuro-Oncology: A Glossary of Terms

**Artificial Intelligence (AI):** A broad field of computer science aiming to create machines that can perform tasks that typically require human intelligence. In neuro-oncology, AI is used to enhance brain tumor diagnosis, prognosis, and treatment.
**Machine Learning (ML):** A subset of AI that enables machines to learn patterns from data and make decisions without explicit programming. ML techniques are widely applied in neuro-oncology for data analysis.
**Deep Learning (DL):** A type of ML using neural networks with multiple layers (deep neural networks). Deep learning is crucial in image and data analysis tasks, contributing significantly to neuro-oncology research.
**Computer Vision (CV):** A field of AI focusing on enabling machines to interpret and understand visual information. In neuro-oncology, computer vision is employed for tasks like medical imaging analysis.
**Large Language Models (LLMs):** Advanced models, like GPT-3, capable of understanding and generating human-like language. LLMs contribute to textual data analysis and interpretation in neuro-oncology.
**Vision Transformers:** Deep learning models specifically designed for image analysis tasks, providing an alternative to traditional Convolutional Neural Networks (CNNs).
**Convolutional Neural Networks (CNN):** A type of deep neural network designed for image recognition and processing. CNNs play a significant role in medical image analysis for brain tumor detection.
**Single Cell RNASeq:** A molecular biology technique that analyzes gene expression at the single-cell level. In neuro-oncology, it aids in understanding the molecular heterogeneity of brain tumors.
**Graph Neural Networks (GNN):** Specialized neural networks for analyzing graph-structured data. GNNs are applied in neuro-oncology to model complex relationships in brain tumor data.
**Explainable AI (XAI):** An approach in AI and ML that emphasizes transparency and the ability to understand and interpret model decisions. In neuro-oncology, XAI is crucial for gaining clinicians' trust in AI-based systems.
**Generative Adversarial Networks (GANs):** ML models comprising a generator and a discriminator, working in tandem to generate synthetic data. GANs find applications in data augmentation and synthesis for neuro-oncology.
**Automated Machine Learning (AutoML):** Tools and techniques automating the process of designing, training, and deploying ML models. AutoML facilitates easier adoption of AI in neuro-oncology by reducing the need for extensive expertise.
**Graphics Processing Units (GPUs):** Hardware accelerators used to enhance the computational power of computers, crucial for training complex neural networks in neuro-oncology.

Field-Programmable Gate Arrays (FPGAs): Reconfigurable integrated circuits used to accelerate specific tasks in AI applications, offering flexibility and efficiency.

Real-time Algorithms: Algorithms designed to provide results or responses within a timeframe that allows immediate action. In neuro-oncology, real-time algorithms facilitate quick decision-making.

Federated Learning: An approach to ML where models are trained across decentralized devices or servers holding local data, ensuring privacy and security in neuro-oncology applications.
